# Should the WHO Growth Charts Be Used in France?

**DOI:** 10.1371/journal.pone.0120806

**Published:** 2015-03-11

**Authors:** Pauline Scherdel, Jérémie Botton, Marie-Françoise Rolland-Cachera, Juliane Léger, Fabienne Pelé, Pierre Yves Ancel, Chantal Simon, Katia Castetbon, Benoit Salanave, Hélène Thibault, Sandrine Lioret, Sandrine Péneau, Gaelle Gusto, Marie-Aline Charles, Barbara Heude

**Affiliations:** 1 INSERM, UMR1153 Epidemiology and Biostatistics Sorbonne Paris Cité Center (CRESS), Early determinants of the child’s health and development Team (ORCHAD), Paris, France, Paris Descartes University, France; 2 Univ. Paris-Sud, Laboratoire de biomathématique, Faculté de Pharmacie, Châtenay-Malabry, France; 3 Université Paris 13, INSERM, UMR1153 Epidemiology and Biostatistics Sorbonne Paris Cité Center (CRESS), Nutritional Epidemiology Research Team (EREN), Paris, France, Paris Descartes University, France, Inra, Cnam, Université Paris 5, Université Paris 7, Bobigny, France; 4 Univ. Paris Diderot, Sorbonne Paris Cité, Paris, AP-HP, Hôpital Robert Debré, Service d’Endocrinologie Diabétologie Pédiatrique et Centre de Référence des Maladies Endocriniennes Rares de la Croissance, Paris, Institut National de la Santé et de la Recherche Médicale, UMR 676, Paris, France; 5 Inserm UMR 1085 IRSET, Rennes, France; Université de Rennes 1, Faculté de Médecine, Rennes, France, Centre Hospitalier Universitaire de Rennes (CHU), Service d’Epidémiologie et de Santé Publique, Rennes, France; 6 INSERM, UMR1153 Epidemiology and Biostatistics Sorbonne Paris Cité Center (CRESS), Obstetrical, Perinatal and Pediatric Epidemiology Research Team (EPOPé), Paris, France, Paris Descartes University, France; 7 Carmen, Inserm U1060, University of Lyon 1, INRA U1235, CRNH Rhône-Alpes, CENS, Lyon, France; 8 Institut de veille sanitaire (InVS), Département maladies chroniques et traumatismes, Unité de surveillance et d’épidémiologie nutritionnelle (USEN), F-94415 Saint-Maurice, France, Université Paris 13, Sorbonne Paris Cité, Centre de Recherche en Epidémiologies et Biostatistiques, Bobigny, France; 9 Univ. Bordeaux, ISPED, Centre Inserm U897-Epidemiologie-Biostatistique, Bordeaux, France, Inserm, ISPED, Centre Inserm U897-Epidemiologie-Biostatistique, Bordeaux, France; 10 French Agency for Food, Environmental and Occupational Health Safety (ANSES, ex-AFSSA, Dietary Survey Unit, Maisons-Alfort, France; 11 IRSA, département Synergies, 37521 La Riche cedex, France; Medical University of South Carolina, UNITED STATES

## Abstract

**Background:**

Growth charts are an essential clinical tool for evaluating a child's health and development. The current French reference curves, published in 1979, have recently been challenged by the 2006 World Health Organization (WHO) growth charts.

**Objective:**

To evaluate and compare the growth of French children who were born between 1981 and 2007, with the WHO growth charts and the French reference curves currently used.

**Design:**

Anthropometric measurements from French children, who participated in 12 studies, were analyzed: 82,151 measurements were available for 27,257 children in different age groups, from birth to 18 years. We calculated and graphically compared mean z-scores based on the WHO and French curves, for height, weight and Body Mass Index (BMI) according to age and sex. The prevalence of overweight using the WHO, the French and International Obesity Task Force definitions were compared.

**Results:**

Our population of children was on average 0.5 standard deviations taller than the French reference population, from the first month of life until puberty age. Mean z-scores for height, weight and BMI were closer to zero based on the WHO growth charts than on the French references from infancy until late adolescence, except during the first six months. These differences not related to breastfeeding rates. As expected, the prevalence of overweight depended on the reference used, and differences varied according to age.

**Conclusion:**

The WHO growth charts may be appropriate for monitoring growth of French children, as the growth patterns in our large population of French children were closer to the WHO growth charts than to the French reference curves, from 6 months onwards. However, there were some limitations in the use of these WHO growth charts, and further investigation is needed.

## Introduction

Growth charts are an essential tool to monitor a child’s development and detect growth disorders in clinical practice (e.g. stunting). In France, the references currently used by clinicians and available in each French child’s health booklet, were derived at the end of the 1970s. These curves were based on growth data of 588 children born in the Paris region, in mid-1950s, and followed-up longitudinally. [1,2] Given the changes in the living conditions since the 1950s, the relevance of these charts for growth monitoring of more recently born children, can be questioned.

The World Health Organization (WHO) recently provided new growth charts, from birth to 5 years [3] and also from 5 to 18 years [4] and countries have been strongly encouraged to adopt these new charts at a national level. In France, there is a lot of variability in growth-monitoring practices, especially in the type of reference growth curves used. [5] The replacement of the French reference curves by those from the WHO could be a first step to standardize growth-monitoring practices. However, epidemiological and clinical consequences of such a replacement need to be evaluated. Differences have already been described between the French references and the WHO growth charts for children under 5 years [6] but no information is available on whether the growth of French children born in recent decades conforms to the WHO growth charts, from birth to 18 years. As recently suggested by the Committee on Nutrition of the European Society for Pediatric Gastroenterology, Hepatology and Nutrition [7], further studies are needed to determine whether the WHO growth charts are appropriate for monitoring growth, and whether they are more appropriate than national reference curves.

Data from French children, born between 1981 and 2007, included in studies in different parts of France, have been pooled for this study. Our first aim was to investigate the position of this population according to both the WHO growth charts and the French reference curves. The growth of exclusively breastfed and never breastfed children were also compared, according to the WHO growth charts. Finally, the prevalences of overweight, obesity and risk of overweight were compared using the WHO growth charts, the French references and the International Obesity Task Force (IOTF) definitions.

## Subjects And Methods

### Background on the WHO growth charts

In 2006, the WHO published new growth charts (www.who.int/childgrowth/en). [3,4] The charts from birth to 5 years were derived from growth data of children from six countries (Brazil, Ghana, India, Norway, Oman, and the United-States), who participated in the WHO-Multicenter Growth Reference Study (MGRS) between 1997 and 2003. [3] Children included in this study were exclusively or predominantly breastfed for at least 4 months and breastfeeding continued to at least 12 months of age, with complementary foods introduced by 6 months of age; mothers had not smoked during or after pregnancy. The WHO decided to extend the curves to 19 years (weight curves are available only up to 10 years), in order to evaluate the growth of school-aged children and adolescents. [4] These extended reference charts were based on existing growth data from North-American children born during the 1960s and the 1970s. These data were combined with the MGRS data to provide smooth curves. In 2014, the Canadian Pediatric Endocrine Group extended the weight curve to 19 years from the same datasets and the same methods than the WHO (http://www.growthcharts.ca). [8] Throughout this article, we will employ WHO growth charts to refer to the 2006 WHO growth charts and its 2014 extension in Canada.

### Children studied

Children born between 1981 and 2007 who had been included in 12 French studies from the general population were analyzed (**Table 1** and **2**). As described in **Table 1** and **2**, depending on the study, weight and height could have been measured, collected or self-reported from child’s health booklets.

**Table 1:** Characteristics of the studies used for analyses of children from birth to 5 years.

Study	Data collection	n (boys/girls)	BirthYears	Years of inclusion	Age range (months)	Study specificity
**EDEN**, [9]	Prospective	994/900	2003–6	2003–6	0–60	Mother-child cohort; multicenter regional survey (two regions); collected, measured data, or self-reported
**ELANCE**, [10]	Prospective	160/119	1983–4	1983–4	0–60	Health centers for children; local survey (Paris area); measured data
**ELFE pilot**, [11]	Prospective	191/151	2007	2007	0–44	Mother-child cohort; multicenter regional survey (four regions); collected or measured data
**EPIPAGE**, [12]	Prospective	287/252	1997	1997	0–60	Controls in a study of children born prematurely (born > 37 weeks amenorrhea); multicenter regional survey (nine regions); collected or measured data
**FLVS**, [13]	Retrospective	175/175	1981–91	1981–91	0–60	Determinants of overweight, following diet intervention program: local survey (two northern cities)(inclusion: from 1981); collected or measured data
**PELAGIE**, [14]	Prospective	734/692	2003–6	2003–6	0–34	Mother-child cohort; regional survey (Brittany); collected or measured data

EDEN, **E**tude des déterminants pré- et post natals du **D**éveloppement et de la santé de l’**E**nfant; ELANCE, **E**tude **L**ongitudinale **A**limentation **N**utrition **C**roissance des **E**nfants; ELFE, **E**tude **L**ongitudinale **F**rançaise depuis l’**E**nfance; EPIPAGE, **E**tude **EPI**démiologique sur les **P**etits **A**ges **G**estationnels; FLVS, **F**leurbaix **L**aventie **V**ille **S**anté; PELAGIE, **P**erturbateurs **E**ndocriniens: étude **L**ongitudinale sur les **A**nomalies de la **G**rossesse, l’**I**nfertilité et l’**E**nfance.

**Table 2:** Characteristics of the studies used for analyses of children from 5 years to 18 years.

Study	Data collection	n (boys/girls)	BirthYears	Years of inclusion	Age range^†^ (years)	Study specificity
**ELANCE**, [10]	Prospective	76/51	1983–4	1984–5	5–20	Health centers for children; local survey (Paris area) (Follow-up duration: 20 years); measured data
**ENNS**, [15]	Cross-sectional	724/732	1989–2004	2006–7	5–17	National survey; randomly selected; measured (98%) or self-reported (2%) data
**FLVS**, [13]	Prospective	173/175	1981–91	1992–7 1999–2003	5–15	Determinants of overweight, following a diet intervention program; local survey (two northern cities); collected or measured data(Follow-up duration: 10 years)
**ICAPS**, [16]	Prospective	477/477	1990	2002	9–20	Physical activity intervention; regional survey (Alsace)(Follow-up duration: 6 years); measured data
**INCA 2**, [17]	Cross-sectional	684/757	1988–2004	2006–7	5–18	National survey; randomly selected; measured data
**INVS/DGESCO**, [18]	Cross-sectional	2067/2040	1990–41997–2001	2000 2007	6–10	National survey; measured data
**IRSA**, [19]	Cross-sectional	1980/2080	1989–2001	2007	6–18	Multicenter regional survey (four regions); measured data
**PNNS en Aquitaine**, [20]	Cross-sectional	5220/5113	1994–2003	2004–9	5–11	Multicenter regional survey (Aquitaine); measured data

DGESCO, **D**irection **G**énérale de l'**E**nseignement **SCO**laire; ELANCE, **E**tude **L**ongitudinale **A**limentation **N**utrition **C**roissance des **E**nfants; ENNS, **E**tude **N**ationale **N**utrition **S**anté; ICAPS, **I**ntervention **C**entered on **A**dolescents’ **P**hysical activity and **S**edentary behavior; INCA 2, Etude **I**ndividuelle **N**ationale des **C**onsommations **A**limentaires; InVS, **I**nstitut de **V**eille **S**anitaire; IRSA, **I**nstitut inter **R**égional pour la **S**anté; PNNS, **P**rogramme **N**ational **N**utrition **S**anté.

^†^age at inclusion for prospective studies

Six longitudinal studies (EDEN, [9] ELANCE, [10] ELFE pilot, [11] EPIPAGE, [12] FLVS [13] and PELAGIE [14]) collected data during the period from birth to 5 years of age. A total of 4830 children (2289 girls and 2541 boys) living in 5 distinct areas of France (**Table 1** and **S1 Fig.**) and born between 1981 and 2007 were included (96% had at least two measurements). Overall, 52940 pairs of weight and height measurements were used.

Information on breastfeeding was available for 2202 children born between 2003 and 2007 from two of these studies (EDEN and the ELFE pilot). A total of 1265 children (592 girls and 673 boys) were exclusively breastfed, 937 (447 girls and 491 boys) were never breastfed. The number of corresponding weight and height measurements was 17174 and 12236 pairs, respectively.

In order to cover the age range from 5 to 18 years, we included data from three longitudinal studies (ELANCE, [10] FLVS [13] and ICAPS [16]) and five cross-sectional studies (ENNS, [15] INCA 2, [17] data from InVS/DGESCO, [18] IRSA, [19] and PNNS en Aquitaine [20]) (**Table 2**). There were 29211 pairs of weight and height measurements from 22457 children (11244 girls and 11213 boys) born between 1981 and the 2004 (6% had at least two measurements).

### Statistical analysis

For each child, age- and sex-specific z-scores were calculated for height, weight and Body Mass Index (BMI), expressed by Standard Deviation (SD), based on the WHO growth charts and on the French references curves. [1,2]. The **L**ambda-**M**u-**S**igma (LMS) method was used to take the asymmetry of the distributions into account. This method summarizes the measurement distributions with three age- and sex- specific parameters, namely: the median (M), the coefficient of variation (S) and the skewness of the distribution (L). A z-score was then calculated as follows: Z = [(y/M) ^L^−1] / LS. If the distribution is symmetric (e.g. for height) then L = 1 and Z = [y-μ]/σ. [21]

The positioning of child growth on the WHO and the French curves was represented graphically, throughout infancy and childhood, separately in girls and boys, using fitted penalized B-spline curves (degree 3) [22] of z-scores regressed on age. Given the distinct WHO growth charts, we separated the periods, from birth to 5 years and from 5 to 18 years.

The same methods were used to compare z-score curves of exclusively breastfed and never breastfed children, based on the WHO growth charts, focusing on the birth to 24 months, period the most susceptible to differ according to breastfeeding status.

The prevalences of overweight (including obesity) and obesity were calculated in both boys and girls according to the WHO growth charts [3,4] and the French references [1] for the various age ranges. The same age groups were used for the IOTF definition, [23] from two years onwards.

All analyses used SAS software (version 9.3; SAS Institute, Cary, NC).

## Results

### Z-scores of growth parameters from birth to 5 years

**Fig 1:**
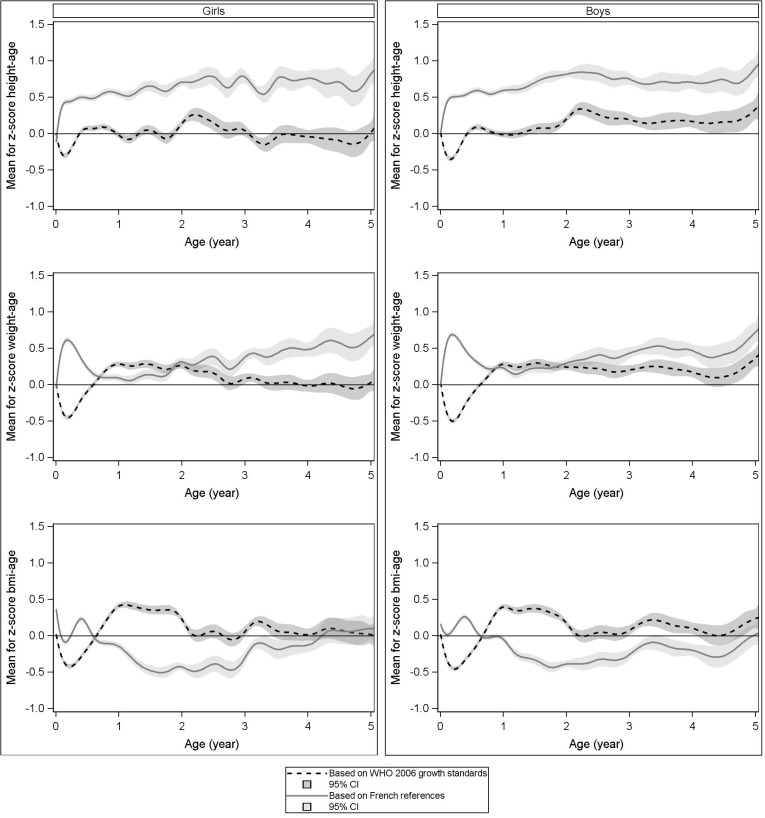
Mean z-scores of our French population (birth—5 years) according to both growth charts.

Mean height, weight and BMI z-scores based on both the WHO growth charts and the French references, for the children in our recently born population, are presented graphically by age (**Fig. 1)**. Z-score curves were very similar across both genders, for all three growth parameters. Mean z-scores for height based on the French references were close to zero at birth but deviated as early as 1 week and until 5 years, illustrating how taller were children from our studied population as compared to the French references (maximal z-score: 0.75SD in girls and 0.80SD in boys, at approximately 2 years and average z-score: 0.5SD in both girls and boys). In contrast, mean height z-scores based on the WHO growth charts showed a trough during the first six months: the nadir was reached at 3 months in girls (-0.30SD) and at 2 months in boys (-0.40SD). After 6 months of age, mean height z-scores based on the WHO growth charts were close to zero.

Weight and BMI z-scores based on the WHO growth charts showed the same pattern. A trough was also observed between birth and 9 months, whereas during this period BMI z-scores based on the French references were close to zero. After 9 months, weight and BMI z-scores based on the WHO curves were positive. As an example, BMI z-scores reached 0.44SD in girls and 0.41SD in boys whereas z-scores based on the French references decreased and reached a nadir at −0.49SD in girls and −0.48SD in boys at 2 years. After 3 years of age, BMI z-scores tended to be close to zero, for both the WHO growth charts and the French references.

### Z-scores of growth parameters from 5 to 18 years

**Fig 2:**
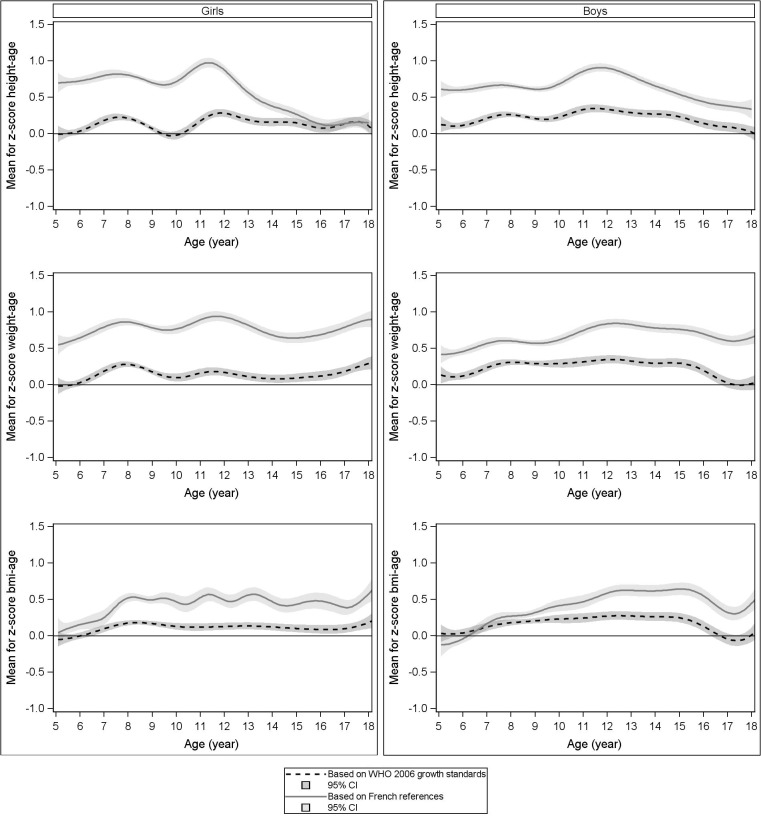
Mean z-scores of our French population (5–18 years) according to both growth charts.

Mean height, weight and BMI z-scores based on the WHO growth charts and the French references are presented graphically by age on **Fig. 2**. Children from our population were taller than the French references, whatever the age. At the end of the pubertal period, mean height z-scores converged however towards the WHO curves in girls. Overall, mean height z-scores were close to zero when based on the WHO growth charts.

Weight z-score curves based on the WHO growth charts or the French references followed the same pattern as height z-score curves. Mean BMI z-scores were null for each reference at 5 years in both genders. Z-scores based on the French references increased thereafter, whereas z-scores based on the WHO growth charts became positive but closer to zero.

### Z-score of growth parameters of exclusively breastfed and never breastfed children

**Fig 3:**
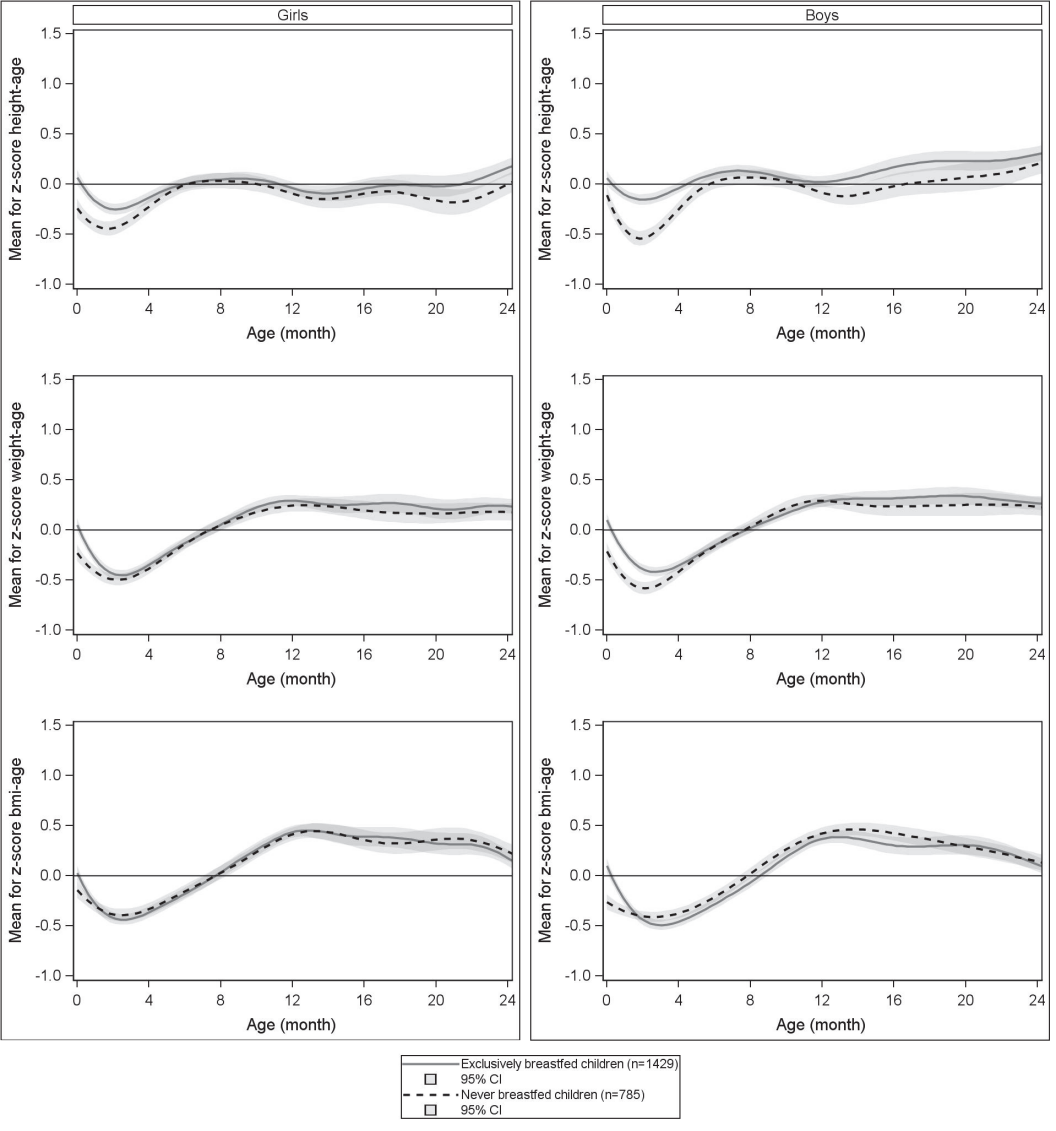
Mean z-scores of exclusively/never breastfed children according to both growth charts.

Mean height, weight and BMI z-scores in exclusively breastfed and never breastfed children based on the WHO growth charts are presented graphically by age on **Fig. 3**. No major difference was observed between the growth patterns of exclusively breastfed and never breastfed children. In particular, both exclusively breastfed and never breastfed children experienced the trough between birth and 6 months, even if this was less pronounced for height in exclusively breastfed children.

### Prevalence of overweight and obesity in children from birth to 18 years

Prevalences of overweight (including obesity), obesity and at risk of overweight defined either according to the French, the IOTF or the WHO curves are shown in **Table 3**. As expected, differences in threshold on references lead to differences in estimates of prevalence. A higher prevalence of overweight was observed with the WHO growth charts, as compared to the French references in girls from 1 to 3 years and in boys from 6 months to 3 years. From 2 to 5 years, in both genders, higher prevalences of overweight were observed with the IOTF references, as compared to both the French and the WHO curves. After 5 years, in both genders, higher overweight and obesity prevalences were observed with the WHO references than with French and IOTF references.

**Table 3:** Percentage of children classified as overweight (including obesity), obese and at risk of overweight.

Age (years)	N	French (≥97th centile)	IOTF (≥C-25)	WHO (>1SD^†^)	WHO (>2SD^‡^)	IOTF (≥C-30)
***GIRLS***		Overweight*	Overweight*	At risk of overweight**	Overweight*	**Obese**
0 to <0.5	*2262*	6.5	-	11.1	1.5	-
0.5 to <1	*2106*	3.8	-	20.0	2.7	-
1 to <2	*1668*	2.5	-	26.3	4.4	-
2 to <3	*1454*	2.1	6.8	16.7	2.8	0.6
3 to <5	*1031*	3.8	8.7	15.8	2.1	1.0
				**Overweight** *	**Obese**	
5 to <8	*3342*	10.6	13.1	16.6	3.9	2.7
8 to <11	*5375*	17.1	17.6	23.5	5.7	3.6
11 to <14	*1283*	13.9	16.3	21.9	4.6	2.5
14 to <18	*2060*	11.6	17.1	18.8	4.4	3.8
***BOYS***		**Overweight** *	**Overweight** *	**At risk of overweight** **	**Overweight** *	**Obese**
0 to <0.5	*2507*	4.4	-	11.8	1.4	-
0.5 to <1	*2298*	3.2	-	20.1	3.6	-
1 to <2	*1773*	2.4	-	26.1	4.6	-
2 to <3	*1599*	2.1	5.6	17.8	2.8	0.4
3 to <5	*1123*	3.8	6.1	17.5	3.0	0.6
				**Overweight** *	**Obese**	
5 to <8	*3531*	7.6	9.9	18.8	5.1	2.4
8 to <11	*5397*	14.1	15.2	25.3	8.7	3.0
11 to <14	*1282*	17.7	20.0	26.9	8.3	3.5
14 to <18	*1830*	13.1	17.2	19.4	5.3	3.9

SD: Standard Deviation; IOTF: International Obesity Task Force

C-25 and C-30 correspond to centiles that match BMI 25 and 30 kg/m^2^ at 18 years

^†^WHO > 1 SD correspond to WHO > 85^th^

^‡^WHO > 2 SD correspond to WHO > 97.7^th^

*Overweight includes ‘obesity’

**At risk of overweight includes ‘overweight’ and ‘obesity’

## Discussion

Growth data from French children, born between 1981 and 2007, from studies in general populations, showed that their growth was closer to the WHO growth charts than to the current French references, except from birth to 6 months of age. Indeed, a striking difference was observed for weight, height and BMI during the first months of life that was not explained by breastfeeding rates.

### From birth to 5 years

The analysis of weight, height and BMI z-scores from birth to 5 years allowed us to highlight subtle differences. An important difference in growth dynamics was observed for weight, height and BMI z-scores during the first six months of life. The trough in the z-score curves during this period indicated a slower growth in French children than expected from the WHO growth charts during the first three months of life, but there was a catch-up at 6 months. This is not a French specificity, since many international studies observed the same z-score pattern compared with the WHO growth charts during the first months of life. [6,24–26] For instance, it was shown that mean BMI z-scores in United Kingdom, Belgian, Dutch, and American children were also lower than the WHO growth charts between birth and 5 months. We and others, showed that breastfeeding did not completely explain this trough, since the same pattern of z-score was observed in exclusively breastfed and never breastfed children. [25,27]

It is worth highlighting that, if the WHO growth charts were used in France, most of the children would be considered as having a slow growth during the first three months of life. In particular, from 4 to 6 months, the prevalence of stunting according to the WHO definition (below −2SD from the median length-for-age) would be about 5% (results shown in **S1 Table**), which is more than twice as high as the expected value of 2.3%. This period is critical for growth monitoring. In fact, when growth is suspected to be too slow, feeding advice is likely to be provided to mothers to promote their child’s growth, as for example, for breastfed children, the introduction of bottle-feeding or even breastfeeding cessation. Studies in physicians showed that the interpretation of a given growth differed according to the reference curves used. [28,29] As discussed by Binns et al., this is a paradox of the WHO growth charts which results in more children being considered as underweight or stunted during the first months of life. This is likely to lead physicians to question breastfeeding continuation more often. This paradox could in part be explained by the population selected for establishing the final growth charts, who represented only a small part of the targeted population. [30]

The current study allowed us to describe how a population of French children, born between the eighties and the 2000s grew in comparison with the French references. The most striking result concerns the children height and weight with a mean z-score close to zero at birth and around 0.5SD as early as one month. This higher weight in young babies, including those who were breastfed, suggests a faster overall growth during the first weeks of life, which could be worrying as a rapid early weight growth has been associated with later metabolic risks. [31] Consistent with our results, other studies showed that French children were taller when born more recently, postnatally [32,33] but not at birth. [34,35] Changes in infant environment, nutrition or care during the past decades may explain this secular trend.

### From 5 to 18 years

From 5 to 15 years, our French population of children was closer to the WHO growth charts than to the French references. After 15 years, height z-scores for girls were close to zero based on to both curves. These results reflected a more rapid height growth in childhood and a faster growth maturation of the French children born recently, compared to the children included in the French references, resulting in a similar final height in adulthood. This was more visible in girls as puberty physiologically ends earlier than in boys. [36,37] It must be noted that the French references were based on a smaller sample (171 children at the end of growth) than the sample assembled for this study, and it is possible that some of the growth patterns were peculiar to this small sample.

### The issue of thresholds for the definitions of overweight and obesity

Obviously, differences in thresholds and references for the definition of overweight and obesity lead to large differences in estimates of prevalence, as pointed out with the illustration of the percentile curves of BMI in girls and in boys according to various references. [38] According to French references, from 5 years of age, overweight prevalence ranged 11% to 17% in both genders, i.e. from 4 to 6 times as high as the expected value of 3%. This illustrates how much French children were more often overweight in 1981–2007 than in the 1950s. It does not however mean that French references for overweight are not appropriate, since it remains relevant to detect more overweight children, if they are indeed much heavier. Based on the WHO growth charts, from 5 years of age, the prevalence of obesity was also higher than the expected value of 2.3%. These higher prevalences (from 5% to 8%) could be explained by the fact that the WHO trimmed obese children from the NCHS to create their growth charts. Our results also show that prevalences differed according to the age range considered, consistent with a previous study. [39] This may be related to secular trend effects. There is however a need to find a consensus for the definition of overweight [38] and other terminologies [40] used for the purpose of international comparisons. Indeed, the methods used to provide thresholds for overweight and obesity are heterogeneous. The IOTF and the WHO curves are a continuum with the adult definition of overweight and obesity (at 18 and 19 years respectively). It allows consistency with the risk of becoming overweight or obese at an adult age. [41] Additionally, compared to the WHO growth charts and the French references, the IOTF references only propose a definition of overweight and obesity from two years onwards. The period spanning from birth to 2 years of age is a time of adjustment between pre- and post-natal growth patterns with a large variability of individual trajectories. [42] Thus, before the age of 2 years, physicians’ evaluations of infant nutritional status is usually based on weight and height separately, rather than on BMI. While the usefulness of overweight categories is questionable at this age, age at adiposity rebound has been identified as a predictor of later obesity. BMI monitoring across childhood should therefore focus on its dynamic dimension as crossing centiles or velocity, especially at this age, as abnormal growth patterns can occur despite a normal absolute BMI. [35]

### Growth charts as a screening tool for stunting

From birth to 18 years, we observed higher prevalence of stunting with the WHO growth charts as compared to French references, especially in the first year of life (results shown in **S1 Table**). We hypothesize that the WHO growth charts will detect more children with an abnormal growth, but may also promote clinical referral for many healthy children. It is worth mentioning that growth charts are just one out of other existing tools for individual growth follow-up, but are especially relevant for monitoring individual growth dynamic. However, further studies are needed to evaluate the consequences of using the WHO growth charts instead of the current French references in terms of sensitivity and specificity in regards to screening for stunting.

### Strengths and weaknesses of our study

Most of the surveys which data come from were not and did not aim to be representative of French children. Potential selection bias could not be formally evaluated, since we did not have information on socio-demographic characteristics for all of them. It should however be stressed that our data cover a large part of the French territory as illustrated on **S1 Fig.** We also performed separate analyses for each survey, and results were consistent across studies (results shown in **S2 Fig.** to **S4 Fig.**), suggesting that any regional or socio-demographic selection biases had little impact on the results and their interpretation. Anthropometric data were either measured or collected from health booklets or self-reported, and are therefore of heterogeneous quality. Data collected from health booklets may be more subject to measurement errors and therefore reduce precision of our estimations. There is however no argument to suggest that these errors would have induced estimation bias, especially since our results were very consistent between studies with distinct methodologies. Importantly, the data from health booklets are appropriate in regards to our main objective, which was to study growth data from usual clinical practice.

### Strengths and weaknesses of the WHO growth charts

The strengths of the WHO growth charts are that they are globally representative, based on six countries and useful for international comparisons, they describe healthy growth, that is, growth as it should be. They correspond with the growth of current French children in the general population. However, the WHO growth charts have limitations: i) the use of two distinct sets of populations (before and after 5 years), ii) a growth pattern suggesting earlier maturation which might not be optimal, and iii) the complex definitions and terminologies of overweight and obesity.

### Conclusion

Growth of recently born French children, from birth to 18 years, appears to be closer to the WHO growth charts than to the French references, except during the first six months of life. Breastfeeding does not seem to explain these differences. The WHO growth charts may be appropriate for growth monitoring of French children, especially for height. However, there are some limitations in the use of the WHO growth charts that require additional study, especially in order to measure the impact of such a change for clinical practice.

## Supporting Information

S1 Fig Data locations of surveys from birth to 5 years. (TIF)

S2 Fig Mean z-scores of French girls from EDEN study (birth – 4 years) according to both growth charts. (TIF)

S3 Fig Mean z-scores of French girls from FLVS study (birth – 3 years) according to both growth charts. (TIF)

S4 Fig Mean z-scores of French girls from Elfe study (birth – 3 years) according to both growth charts. (TIF)

S1 Table Percentage of children classified as stunted according to both growth charts. (PDF)
