# Supplementary figures and images for: Should the WHO Growth Charts Be Used in France?
Source: PLoS One. 2015 Mar 11;10(3):e0120806. doi: 10.1371/journal.pone.0120806 (PMC4356547; doi:10.1371/journal.pone.0120806)

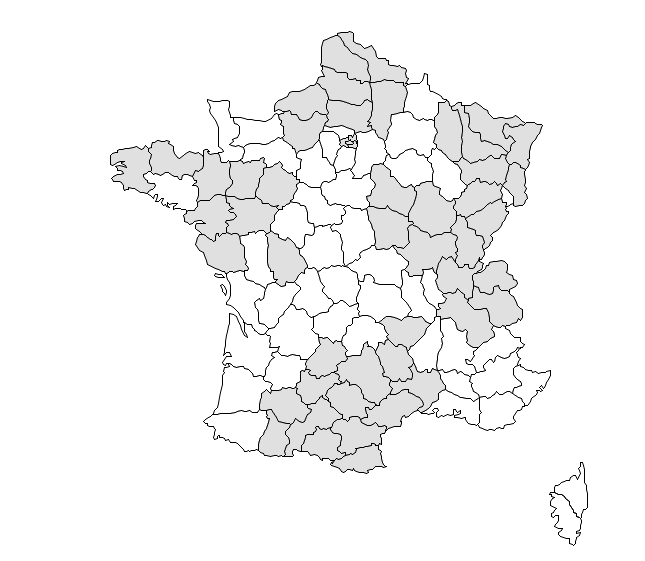

Supplement: S1 Fig — (TIF) [file pone.0120806.s001.tif]

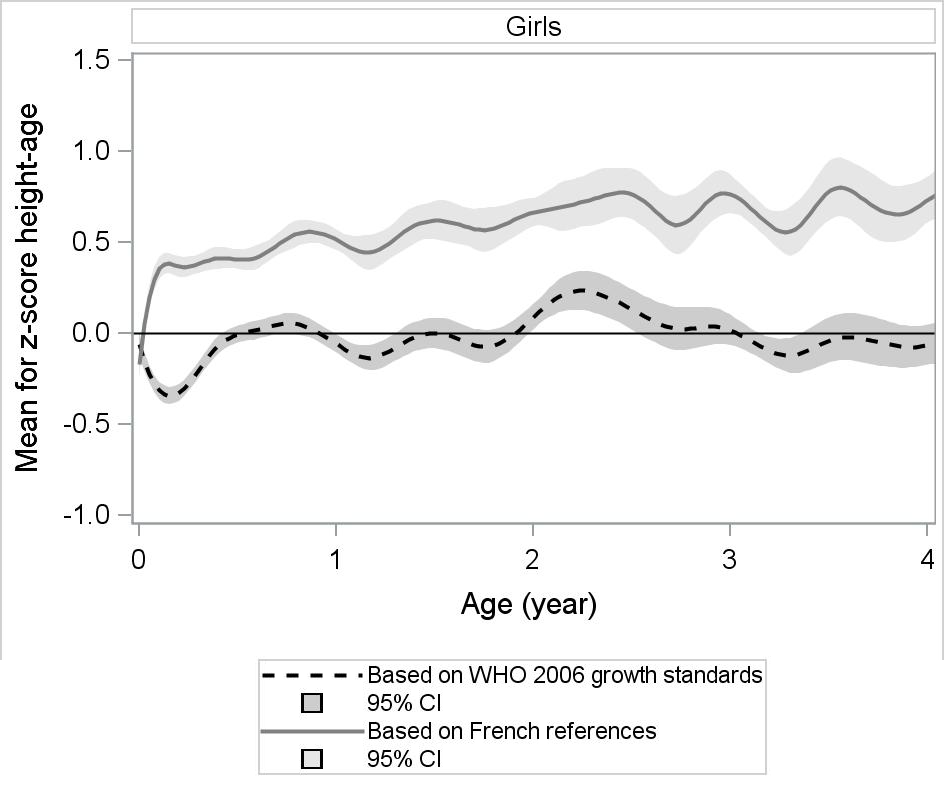

Supplement: S2 Fig — (TIF) [file pone.0120806.s002.tif]

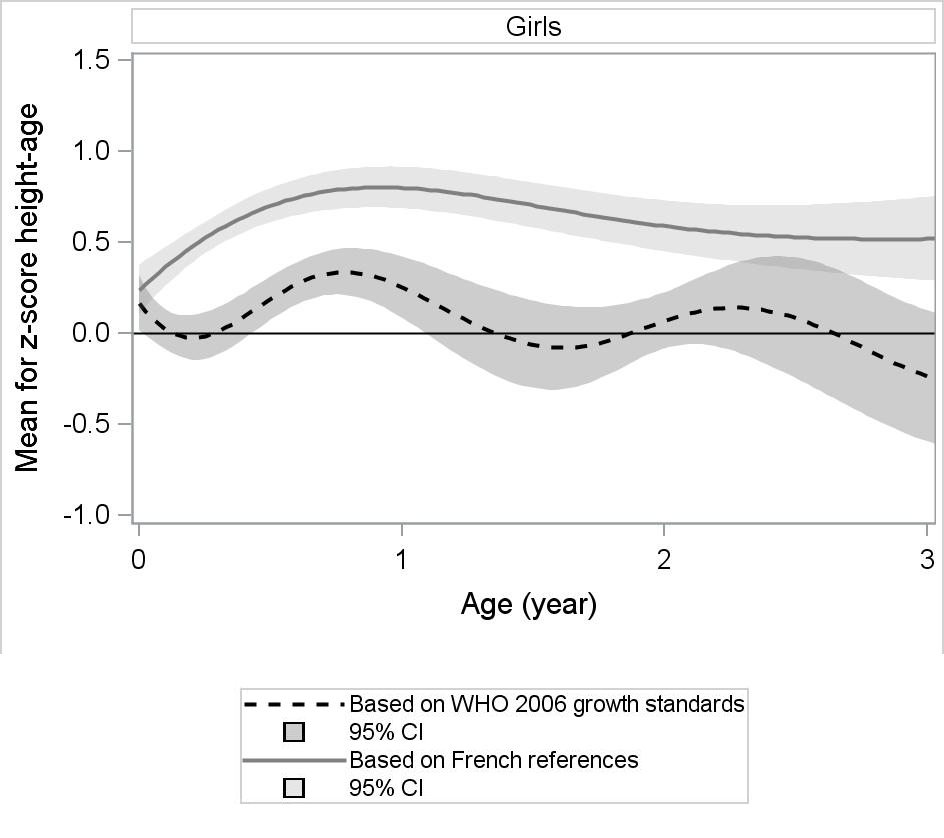

Supplement: S3 Fig — (TIF) [file pone.0120806.s003.tif]

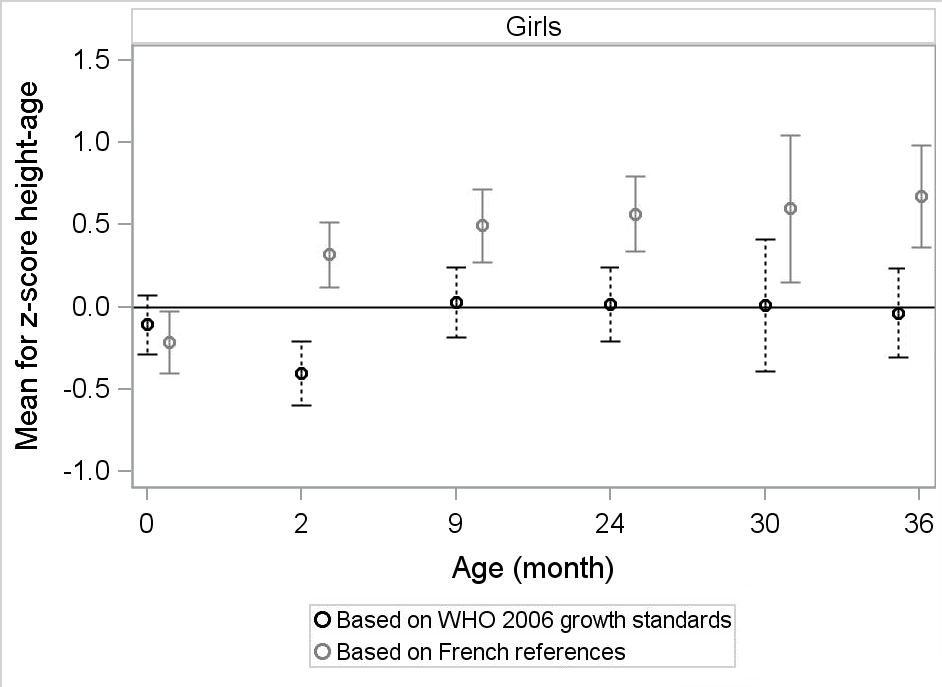

Supplement: S4 Fig — (TIF) [file pone.0120806.s004.tif]
