# Supplementary material for: Should the WHO Growth Charts Be Used in France?
Source: PLoS One. 2015 Mar 11;10(3):e0120806. doi: 10.1371/journal.pone.0120806 (PMC4356547; doi:10.1371/journal.pone.0120806)
Supplement: S1 Table — (PDF) [file pone.0120806.s005.pdf]

S1 Table. Percentage of children classified as stunted according to both growth charts.

Age (year)	n	FR (< 3th)	WHO (< $2SD^{\dagger}$)
GIRLS			
0 to <0.5	*2262*	3.6	5.2
0.5 to <1	*2106*	0.9	2.1
1 to <2	*1668*	1.3	3.2
2 to <3	*1454*	0.6	1.0
3 to <5	*1031*	0.7	1.7
5 to <8	*3342*	1.0	1.8
8 to <11	*5375*	1.2	1.7
11 to <14	*1283*	1.6	1.8
14 to <18	*2060*	2.3	0.9
BOYS			
0 to <0.5	*2507*	2.2	5.5
0.5 to <1	*2298*	1.3	3.4
1 to <2	*1773*	0.7	3.0
2 to <3	*1599*	0.5	1.4
3 to <5	*1123*	0.3	1.3
5 to <8	*3531*	0.7	1.3
8 to <11	*5397*	1.0	1.1
11 to <14	*1282*	0.9	1.9
14 to <18	*1830*	1.7	1.3

SD: Standard Deviation;
† WHO < 2 SD correspond to WHO < 2.3^{th}
